# Health and longevity studies in *C. elegans*: the “healthy worm database” reveals strengths, weaknesses and gaps of test compound-based studies

**DOI:** 10.1007/s10522-021-09913-2

**Published:** 2021-03-08

**Authors:** Nadine Saul, Steffen Möller, Francesca Cirulli, Alessandra Berry, Walter Luyten, Georg Fuellen

**Affiliations:** 1 Molecular Genetics Group, Institute of Biology, Humboldt University of Berlin, 10115 Berlin, Germany; 2 Institute for Biostatistics and Informatics in Medicine and Ageing Research, Rostock University Medical Center, 18057 Rostock, Germany; 3 Center for Behavioral Sciences and Mental Health, Istituto Superiore di Sanità, 00161 Rome, Italy; 4 Department of Biology, KU Leuven, 3000 Leuven, Belgium

**Keywords:** Database, *C. elegans*, Aging, Compounds, Phenotypes, Healthspan

## Abstract

Several biogerontology databases exist that focus on genetic or gene expression data linked to health as well as survival, subsequent to compound treatments or genetic manipulations in animal models. However, none of these has yet collected experimental results of compound-related health changes. Since quality of life is often regarded as more valuable than length of life, we aim to fill this gap with the “Healthy Worm Database” (http://healthy-worm-database.eu). Literature describing health-related compound studies in the aging model *Caenorhabditis elegans* was screened, and data for 440 compounds collected. The database considers 189 publications describing 89 different phenotypes measured in 2995 different conditions. Besides enabling a targeted search for promising compounds for further investigations, this database also offers insights into the research field of studies on healthy aging based on a frequently used model organism. Some weaknesses of *C. elegans*-based aging studies, like underrepresented phenotypes, especially concerning cognitive functions, as well as the convenience-based use of young worms as the starting point for compound treatment or phenotype measurement are discussed. In conclusion, the database provides an anchor for the search for compounds affecting health, with a link to public databases, and it further highlights some potential shortcomings in current aging research.

**Supplementary Information:**

The online version contains supplementary material available at 10.1007/s10522-021-09913-2.

## Introduction

Why do we age, why does our health tend to deteriorate with age, and how can we affect the underlying processes in a positive way? Such questions are raised by numerous biogerontologists and physicians worldwide, with already quite specific and applicable answers. We already know that the rate of aging is determined by our genomic constitution in combination with environmental factors, such as nutrition or physical activity, in addition to epigenetic patterns, which represent the interface between them (Adwan-Shekhidem and Atzmon 2018; Benayoun et al. 2015). While our transcriptional, genetic and epigenetic landscapes are already useful to predict the expected lifespan and the rate of aging of an individual (Bell et al. 2019; Kudryashova et al. 2020), it is difficult to exert a direct influence on these. Thus, environmental factors (above all nutrition) are a primary target for people to combat aging. This is reflected in the increasing number of nutritional supplements targeting aging and age-associated diseases.

The growing number of papers reporting on promising lifespan-extending compounds in model organisms is not only encouraging, but also making it difficult to select the most suitable candidate compounds for further evaluation in humans. For this, databases are very helpful, and numerous aging-related databases are already available. This abundance (for an overview of available databases see Online Resource ESM_1) could provoke the question as to why is there still a need for a new biogerontology database as presented in this paper. First of all, most databases pertain to genetic analyses, which can be mainly used for prognostic tasks but hardly (so far) for suggesting treatments. Data collections focusing on compound interventions are rare; only AgeFactDB, DrugAge, and Geroprotectors offer compound-treatment results in different model organisms (Online Resource ESM_1). All experimental data collected in these databases deal with survival data, measuring lifespan. However, aging not only triggers an increased risk of mortality, it also causes a decrease of health (defined by disease and dysfunction, see below), specifically a decline in physiological, physical, cognitive, and reproductive function (Fuellen et al. 2019). Given that most people do not care that much about how long they will live, but more about how long they will remain in good health and capable to deal with daily activities in an autonomous way, compounds promoting health, and specifically preventing cognitive and physical decline, are of special interest. Notably, an increase in lifespan is not automatically linked to an increase in healthspan (Hansen and Kennedy 2016).

We defined health, as precisely as possible, as the period of life in which an individual is healthy, that is, doing better than average (in comparison to a reference population) in terms of disease and dysfunction (Fuellen et al. 2019). Our definition of health (and healthspan) was motivated by definitions published before, e.g. Luyten et al. (2016), and we operationalized the various aspects of dysfunction based on the literature, yielding physiological, physical, cognitive, and reproductive function (see above; we ignore all aspects of disease when referring to animal models). In particular, we suggested that stress resistance is a feature of health reflecting physiological function, and (stimulated) locomotion is a feature of health that integrates physical & cognitive function, while pharyngeal pumping in *Caenorhabditis elegans* (*C. elegans*) reflects physical function only. In earlier experimental work, Bansal and colleagues (2015) could show that long-lived *C. elegans* mutants feature an increased proportion of “unhealthiness” during their lifetime, determined by measuring locomotion, stress resistance, pharyngeal pumping, and autofluorescence. However, Hahm et al. (2015) reanalyzed the same dataset by using a different normalization method, leading to different results with various healthspan improvements in *daf-2* mutants. Nevertheless, a missing positive correlation between lifespan and healthspan was observed in lamotrigine-treated flies: lamotrigine increased longevity in parallel with impaired health (based on locomotion and the metabolic rate determined by CO_2_ production) in *Drosophila melanogaster* (Avanesian et al. 2010). Moreover, examples were also reported in vertebrates as Garcia-Valles et al. (2013) and Mitchell et al. (2018) provided evidence for treatments to improve healthspan but not lifespan in mice: age-related decrease in strength, endurance and motor coordination was prevented by life-long exercise (Garcia-Valles et al. 2013) and chronic nicotinamide supplementation resulted in an improvement of glucose homeostasis in mice on a high-fat diet, and prevented hepatosteatosis, inflammation, and oxidative stress in the liver (Mitchell et al. 2018), whereas no lifespan changes were visible in either of the two studies. From a human perspective, focusing on healthspan instead of lifespan is becoming more and more urgent due to the increase of aging related diseases and dysfunctions which accompany the increase in life expectancy (Crimmins 2015; Olshansky 2018; Olshansky and Carnes 2019). Although the increase in lifespan over the past decades has also added years in good health, the percentage of one’s lifetime spent in poor health appears not to have decreased, and may even have increased somewhat. In absolute number of years, we therefore spend more years in ill health the longer we live. There is therefore room for morbidity compression, whereby the fraction of one’s life spent in ill health would decrease. Although lifespan and healthspan are often correlated, there are cases where they are differentially affected as described above.

Thus, in addition to lifespan-related data collections, there is a need for databases with a focus on healthspan, which can enable the selection of potential healthspan-promoting compounds for future medical intervention. Here we present the “Healthy Worm Database” (http://healthy-worm-database.eu), a collection of healthspan-related compound intervention studies in *C. elegans*. Besides flies and rodents, this nematode is one of the most popular model organisms in aging studies (Shen et al. 2018; Son et al. 2019). Furthermore, *C. elegans* offers numerous human disease-specific strains (Markaki and Tavernarakis 2020) and is frequently used to pre-screen compounds for their later pharmaceutical usage (Chen et al. 2015; Dengg and van Meel 2004; Matsunami 2018; Papaevgeniou and Chondrogianni 2018) and also reviewed in Kim et al. (2017). An additional systematic review focusing on healthspan in rodents was recently published (Musillo et al. 2021).

Besides the selection of interesting compounds through the “Healthy Worm Database”, we were able to uncover strengths and weaknesses of *C. elegans-*based aging studies. The challenges and problems in performing lifespan-studies in *C. elegans* were already described in detail by Gruber et al. (2009). However, due to the complex nature of healthspan, healthspan studies are even more difficult to perform and interpret, as we will demonstrate based on our database.

## Materials and methods

### Search strategy

**Fig. 1:**
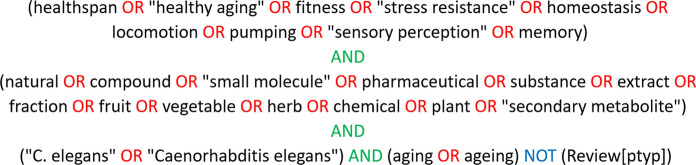
Literature query. The search phrase used in NCBI PubMed to collect relevant literature is shown

A search in PubMed (https://www.ncbi.nlm.nih.gov/pubmed) was performed in January 2019 and for an update in December 2020 to identify relevant studies examining the effects of natural and synthetic compounds, as well as combinations of compounds and extracts affecting healthspan. The target organisms of our search was the model organism *C. elegans* and the target phenotypes are based on Fuellen et al. (2019), who grouped the aging-related phenotypes as follows: (i) physiological, (ii) physical, (iii) cognitive and iv) reproductive function. Thus, a systematic search was performed by carefully selecting terms (Fig. 1) yielding a high hit rate in these aging-relevant parameters. Several search phrases were created and tested, until the optimized version, resulting in a manageable number of hits, was found. Since the term “health” led to too many unspecific results, it was replaced by “healthspan”, “healthy aging” and “fitness”, which improved the quality of the outcome. Of note, longevity or lifespan were not included among our keywords, since this study is focusing on healthspan in clear separation from lifespan, for which we refer to the DrugAge database (Barardo et al. 2017). However, lifespan data were included in our database if these were mentioned in the true positive hit papers. The same strategy was applied for reproductive function, which was not covered by the search-term due to lack of relevance for the health problems of aged human populations. Nevertheless, if data dealing with reproductive function were mentioned in the selected publications, they were added to the database since reproductive organs continue to play a role in the health of elderly people (e.g. male impotence, prostate enlargement, uterine prolapse, cancers of the reproductive organs, etc.). Furthermore, data concerning healthspan biomarkers, as well as supposed interactions of the retrieved compound with genes, proteins, or pathways, were recorded in addition.

### Selection of relevant literature

The papers retrieved were subjected to a second selection process (see Online Resource ESM_4), where additional inclusion and exclusion criteria are described. All paper abstracts were manually checked according to these criteria to confirm the relevance of the retrieved items. Furthermore, 22 papers, which were not detected by the search phrase, were additionally selected due to the relevance of the findings reported. The reasons for excluding papers which were retrieved by the search phrase are collected in the Online Resource ESM_5. Compounds which were only tested for lifespan were added to the database, as long as they were published along with a compound studied for healthspan changes, since the confidence in the healthspan-related findings increases if the same study confirms prior findings on another drug.

Data from the finally selected papers were entered in the database, which includes the compound name (including PubChem ID), full literature reference, conditions (concentration, exposure time and drug regimen, food, growth medium, additives, temperature, and strain), and the observations. The latter were denoted as the change in comparison to the untreated control (increased, decreased, no change, or variable effect, that is, “up”, “down”, “none”, or “variable”, respectively) of the relevant phenotype, including a significance measure (p < 0.05: yes, no, n.d., or variable) and notes, if applicable, which provide additional details about the age of the worms during the test or other performance details. Finally, provided that absolute or percentage values were given, the percentage changes were copied or calculated, and entered into the database. Recently, the blinding status of collected studies was added, which enables the evaluation of the objectivity during phenotypic measurements.

### Analyses and data structure

All collected data are freely available online, and data can be added/edited/amended/corrected upon request or in collaboration. The node.js web interface offers URLs for dynamically created exports as JSON—or tab-delimited files, for downstream analyses with Excel or R (https://stat.ethz.ch/pipermail/r-help/2014-October/422975.html), with which the analyses for this paper were performed.

The data are organized as a table with one row per condition and measured phenotypes in columns. Every line concludes with a reference to the paper from which the information was retrieved. The same condition in another paper warrants another line. If the paper found an effect on the transcriptome, proteome or metabolome, then this is also summarized in a separate column. Supposed interactions of the compound with genes, proteins, or pathways are given in the column “targets”. Phenotypes are represented as quadruplets that indicate the direction of a change, the effect size in percent, the reported p-value, and a comment as free text.

## Results

### Database structure and content

**Fig. 2:**
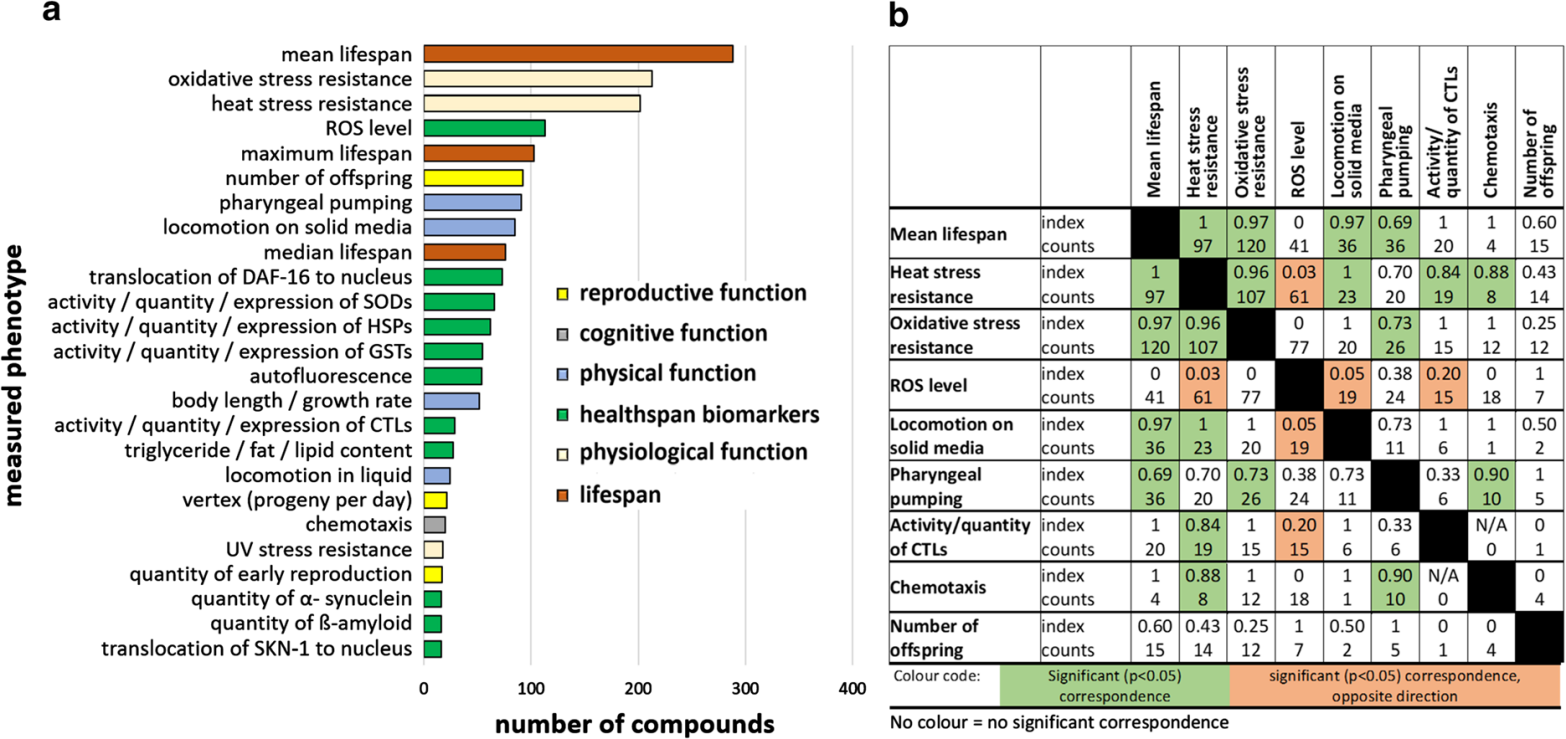
Analyzed phenotypes in *C. elegans* aging studies. The published phenotypes per compound were added up over all database entries. The phenotypes measured for at least 16 compounds are displayed (**a**). The color of the respective bars represents the allocation to one of the six phenotype groups as shown in the legend. Furthermore, the Jaccard similarity index between phenotypic measurements was calculated and a selection of the most used phenotypes and their correspondence with each other is shown (**b**). The counts represent statistically significant measurements and the index refers to the Jaccard similarity index. The significance of correspondence between two phenotypes was determined using the chi square test. Green colored cells indicate a significant (p < 0.05) correspondence between two phenotypes and a significant correspondence in opposite directions is marked red. Note: The Jaccard similarity index was already analyzed in January 2019, thus, excludes publications from 2019 and 2020

When performing data analyses for this study (December 20th 2020), our database referred to 89 phenotypes belonging to six phenotype groups related to health (Fig. 2a), 440 compounds (of which 242 have a PubChem ID assigned), 189 different scientific papers, and 2995 different conditions, which are based on the compound that was administered and its concentration, the exposure time and drug regimen, as well as the food given during the experiment, growth medium and additives, temperature, and the *C. elegans* strain. The change of a phenotype due to the respective compound treatment under a certain condition is described based on performance in a set of health-related measurements (Online Resource ESM_2). This description is provided qualitatively in the columns “Change compared to control” (increases are given as “up”, decreases as “down”, unchanged parameters are given as “none”, and “variable” indicates mixed responses), and quantitatively as a percentage value compared to control, if available. In addition, the significance level (considering the standard p < 0.05 threshold) as well as performance details and time points of test performance, if applicable, are stated. In a few cases, some information like the age of the nematodes during test performance or treatment-initiation are only vaguely indicated by the authors; then, the data were determined based on a best estimate. Finally, the reference is given for each tested condition. To enable a targeted search of relevant information, the entries can be filtered according to the strain used or the observed phenotype. Moreover, a search for a particular compound is possible. All entries are downloadable into a single combined table (Online Resource ESM_3), which can be used for specific analyses. This also allows specific search options, such as finding all compounds tested in combination with UV-killed bacteria, or tested in at least four different concentrations or at concentrations in the nanomolar range, or finding compounds tested in liquid medium, etc.

### Cognitive functions are widely neglected in *C. elegans* health studies

Papers selected in our literature search (see Materials and Methods for detailed search strategy) are completely represented with all their data on health and survival. Thus, despite focusing on health, (mean) lifespan was often included in the database by “piggybacking”, and it turned out to be the most studied phenotype in the database (Fig. 2a), followed by resistance to oxidative- and heat stress. Apart from features describing health-related function, molecular biomarkers of health, called “healthspan biomarkers”, were measured alongside the functional data, and included in the database. Most of these relate to the abundance or activity of a certain protein (e.g.DAF-16 or SKN-1) and its localization, or of gene/protein classes such as heat shock proteins (HSPs), superoxide dismutases (SODs), glutathione S-transferases (GSTs) and catalases (CTLs). The most common molecular “healthspan biomarkers” not directly referring to a gene or protein were reactive oxygen species (ROS), autofluorescence and triglyceride/fat/lipid content.

Considering how often a particular aging-related measurement is performed, the most striking feature highlighted by Fig. 2a is that cognitive function is almost absent among the studied phenotypes. Only chemotaxis, which is a measurement of sensory perception, was occasionally investigated, but memory, neuronal survival/integrity or mechanosensation (also a measurement of sensory perception), were only determined for less than 10 compounds (which are not shown in the figure but are available in supplementary Online Resource ESM_2). Since *C. elegans* is a suitable organism to study aging-related cognitive decline (Stein and Murphy 2012), and since compound-mediated inhibition of that decline in humans is of major interest, this is an astonishing finding at first glance. Nevertheless, it needs to be noted that cognitive function is also necessary for locomotion, in particular when locomotion was provoked by an external stimulus (Fuellen et al. 2019). Thus, this phenotype should be (partly) covered by locomotion measurements.

The small number of cognitive tests in *C. elegans* compound-based aging studies might result from the paucity of routine screening platforms, and the generally limited use of cognitive tests in all areas of *C. elegans* studies. This could be explained by the rather complicated nature of the tests, combined with a relatively high rate of variation compared to other measurements and therefore, restricted reproducibility (unpublished observations). One could argue that the presence of only 302 neurons in *C. elegans* are just not enough to perform complex tasks, such as “associative learning” or “cognition”. Nevertheless, *C. elegans* shows associative and non-associative behavioural plasticity, as well as the ability to form both short-term and long-term memory (Ardiel and Rankin 2010; Arey and Murphy 2017; Cerutti and Levin 2006).

Furthermore, it needs to be noted that measurements such as “body length/growth rate” and “vertex (progeny per day)” are not part of the original healthspan-definition as described in Fuellen et al. (2019). However, growth and reproductive parameters are often used to determine trade-offs of health- and lifespan prolonging treatments (Gruber et al. 2007; Saul et al. 2013; Van Voorhies et al. 2006) and thus, they provide important additional information, which justifies their inclusion in this database.

### Weak correspondence of pharyngeal pumping rate with other healthspan phenotypes

To describe the degree of correspondence of phenotypes, the Jaccard similarity index (Real and Vargas 1996) was calculated for all pairs of phenotypes. This describes the fraction of experiments (across all strains, compounds and concentrations) for which two phenotypes are changed in the same or opposite direction. A value of 1 indicates a perfect correspondence. Data points were included as long as any significant qualitative change (increase or decrease) was determined in both phenotypes. Figure 2b lists a selection of the most-used phenotypes, and their correspondence with each other. The counts represent all statistically significant measurements, and colored cells indicate a significant (p < 0.05) correspondence between two phenotypes. P-values were determined using the chi square test, only considering experiments for which significant changes in either direction are reported in the database. No p-value was determined for pairs of phenotypes that have not been studied together, or for which both phenotypes show no variation in their change across all experiments. Finally, the color distinguishes an observed significant correspondence with a Jaccard similarity index > 0.5 (marked green) and an observed significant correspondence in opposite direction with a Jaccard similarity index < 0.5 (marked red), whereas an index = 0.5 represents an equal distribution of phenotypic changes.

Interestingly, heat and oxidative stress resistance, as well as locomotion, showed strong correspondences with each other, and also with mean lifespan. Furthermore, the level of endogenous oxidative stress, indicated by ROS level, is strongly negatively linked with heat stress resistance and locomotion. Nevertheless, other health parameters seem to be more independently regulated: pharyngeal pumping corresponded only slightly with the aforementioned phenotypes, with Jaccard similarity indexes between 0.69 and 0.73. Due to the relatively low fraction of chemotaxis and reproduction assays, significant correspondences with these phenotypes are rare. Nevertheless, chemotaxis is positively linked with heat stress resistance and pharyngeal pumping. Moreover, the number of offspring is slightly negatively linked with stress resistance, although this does not reach statistical significance. This observation might be a further argument to support the idea of a trade-off between reproduction and lifespan or stress resistance (Aguilaniu 2015; Aprison and Ruvinsky 2014). However, more data are needed to support this conclusion.

### Compound treatments and phenotypic measurements in very young adults and larval stages

**Fig. 3:**
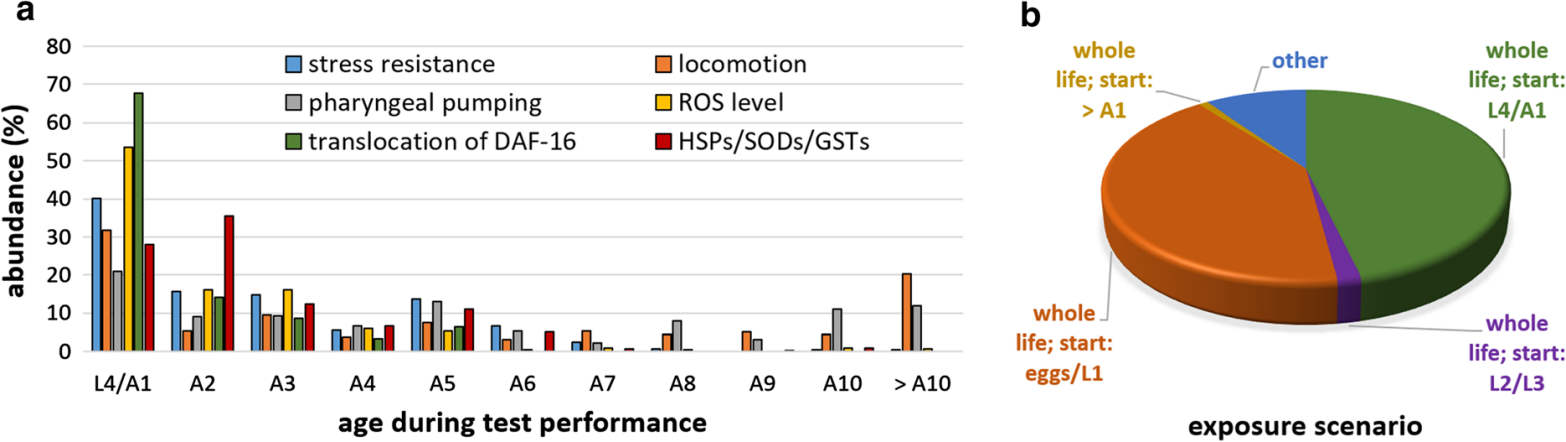
Nematode’s age at time of test performance and exposure initiation. The age of the nematodes at the day of test performance (or test initiation, respectively, for survival-based tests), were summarized for assays analyzing stress resistance (including heat, oxidative, pathogenic, UV, and osmotic stress resistance), locomotion (on solid and in liquid media), pharyngeal pumping, ROS level, translocation of DAF-16, and HSPs/SODs/GSTs (including the activity, quantity or expression of these genes c.q. proteins). The percentage distribution was calculated according to the total number of age groups investigated per phenotype (**a**). In addition, the most popular exposure periods to treat the nematodes with the test compounds were determined. The graph represents the distribution of exposure scenarios used in the collected studies in the database (**b**). A1-A10 = 1st–10th day of adulthood; L1-L4 = 1st–4th larval stage

Another salient feature of worm health- and aging studies was found by summarizing the age classes on the day of test performance for some of the most common phenotypes. The vast majority of phenotypic assays was performed in very young nematodes between the last larval stage and the 3rd day of adulthood (Fig. 3a). A few tests were also performed between the 4th and 6th day of adulthood, but later ages were hardly used. Only physical function, i.e. pharyngeal pumping and locomotion, were occasionally investigated in older worms, even after the 10th day of adulthood. Since the studies collected in the database are mainly dealing with aging, it is surprising that aged nematodes are not the main target of the investigations.

Furthermore, the timing of the start of compound treatment is a crucial factor for the performance of healthspan assays. In 46% of the studied conditions, the exposure to the test compounds started in the last larval stage (L4) or in the first adult stage (A1) (Fig. 3b). Treatment initiation in the egg-stage or the first larval stadium (L1) was performed in 42% of the trials, whereas the other larval stages were hardly used as starting points, perhaps because they are difficult to identify and time. In ~ 10% of the experiments, other exposure scenarios were used, including very short or repetitive exposure, or exposure which was discontinued at a certain age. Overall, only 1% of the studies were performed with exposure starting at older ages (older than A1) (Fig. 3b).

### Live OP50 is the most frequently used food in *C. elegans* compound treatment studies

**Fig. 4:**
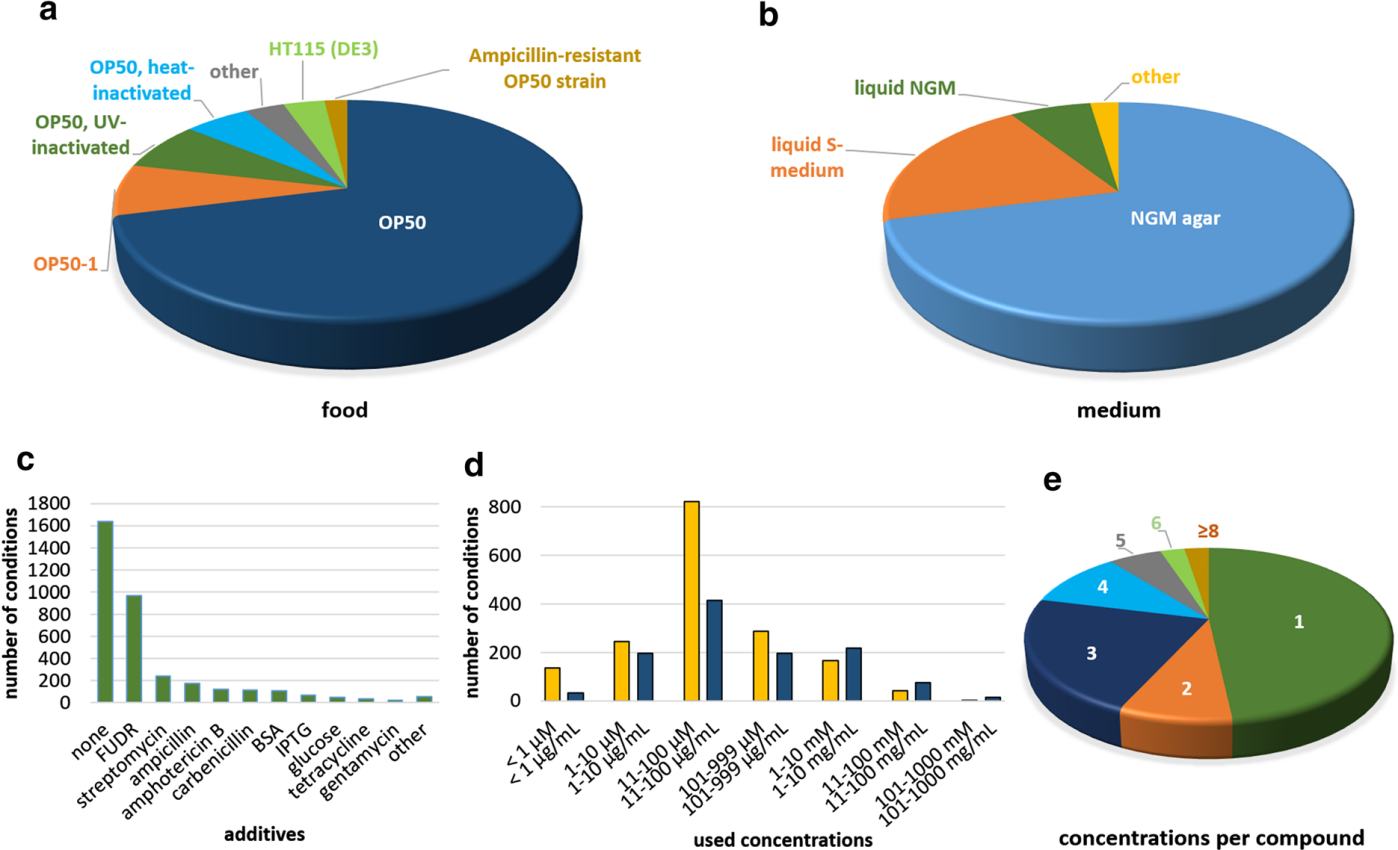
Most popular conditions in *C. elegans* aging studies. The most commonly used food (**a**), growth medium (**b**) and additives (**c**) collected from all studies included in the “Healthy Worm Database” are displayed. Moreover, the most applied concentrations in a molar (yellow) or mass per volume range (blue) are also visualized (**d**), as well as the number of tested concentrations per compound (**e**)

**Fig. 5:**
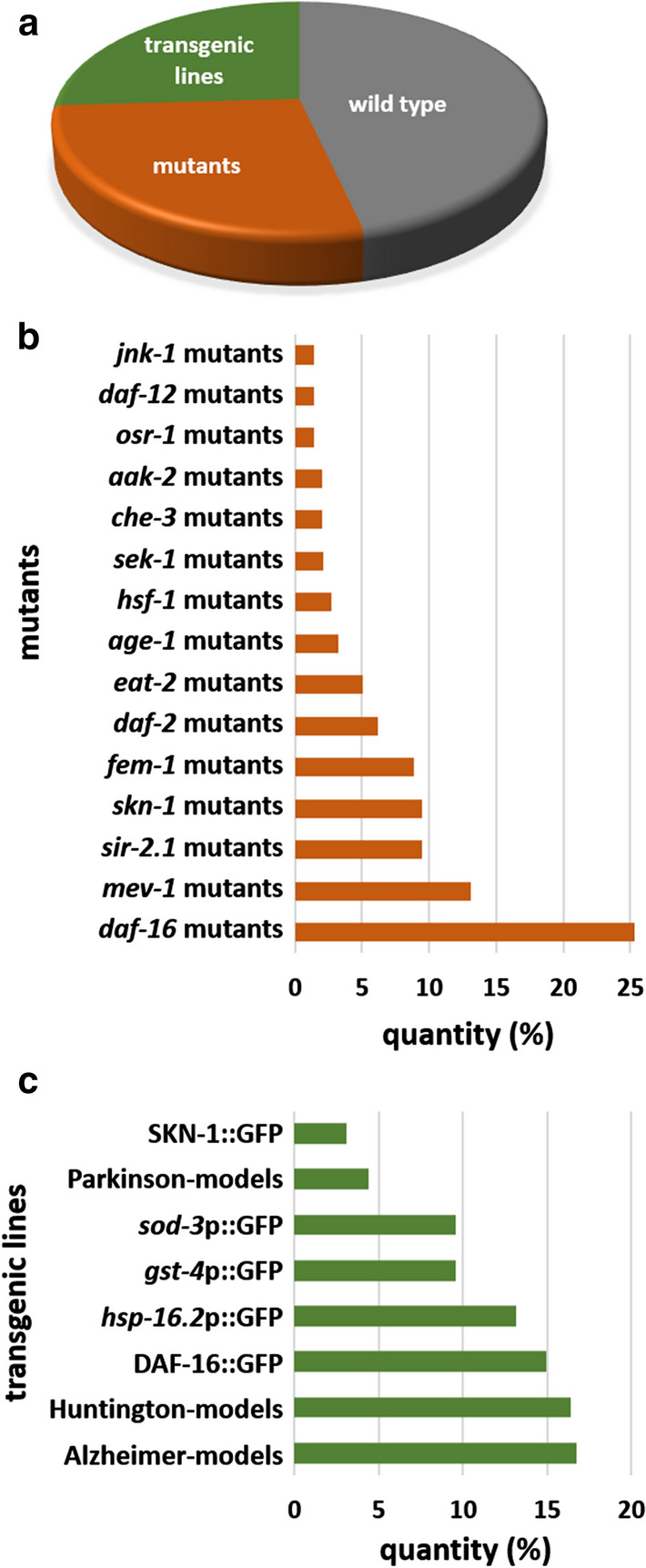
Use of *C. elegans* strains. The distribution of *C. elegans* strains among all conditions is shown (**a**). Furthermore, the most used mutant (**b**) and transgenic (**c**) strains are displayed. The quantity is given in percent from a total of 830 conditions with mutant involvement (**b**) or 775 conditions with transgenic strains (**c**)

*C. elegans* was already introduced as a model organism in the 1960s, and the simple handling of the organism was described in detail (Brenner 1974). This protocol seems to be still the basis for most of the worm labs, as reflected in Fig. 4a and b. About 71% used standard Nematode Growth Medium (NGM) agar plates (Fig. 4b). The solid NGM plates are advantageous in manually performed tests, due to the easier transfer and observation of the worms; thus, liquid media are mainly used in automated assays. The outcome of phenotypic measurements could be influenced by the selection of a solid or liquid medium. For instance, Houthoofd et al. (2002) observed higher enzyme activities in worms grown on solid medium compared to those in liquid medium. They assume that different oxygen concentrations might be the reason for this discrepancy. About two-thirds of the studies were performed with live OP50, the standard *Escherichia coli* feeding strain (Fig. 4a), whereas heat- or UV-inactivated bacteria were only used in 13% of the studies collected in the database. Antibiotic resistant strains, such as OP50-1, are typically used together with streptomycin, which is the second most common additive in the database (Fig. 4c). Interestingly, one-third of the conditions include treatment with fluorodeoxyuridine (FUdR) (Fig. 4c). This inhibitor of DNA synthesis suppresses the reproduction of *C. elegans*, and thus is an important and sometimes indispensable tool, especially for larger or automated compound screenings. However, FUdR was shown to have a (mainly positive) influence on the life- and healthspan of *C. elegans* (Angeli et al. 2013; Wang et al. 2019), and is itself part of the “Healthy Worm Database”. Kato et al. (2017) suggest that FUdR influences lifespan “by interfering with fertility, which extends lifespan, and by inducing DNA base damage, which reduces lifespan”. Furthermore, FUdR was shown to influence hormetic responses (Anderson et al. 2016), proteostasis (Angeli et al. 2013; Feldman et al. 2014), the lifespan and metabolism of certain mutant strains (Aitlhadj and Stürzenbaum 2010; Davies et al. 2012; Van Raamsdonk and Hekimi 2011), as well as polyglutamine aggregation in a Huntington's disease model (Brunquell et al. 2014). Therefore, to prevent potential false-negative or false-positive effects, its use should be avoided, if possible. Furthermore, in numerous studies, FUdR is used in lifespan determination or stress survival tests, but not in any other phenotypic measurement (Online Resource ESM_3), which complicates the direct comparison of phenotypes between conditions including and excluding FUdR. Another possibility to keep synchronicity in worm populations is to use temperature-sensitive germ-line defective mutants, such as the strains BA17 (deficient in *fem-1*), which was only used in 63 of the 2995 conditions studied in our database (Fig. 5b), or SS104 (deficient in *glp-4*), which only occurs four times in our database (Online Resource ESM_6). Additional strains were also described by Fabian and Johnson (1994). The different ways of keeping synchronous cultures and their advantages and disadvantages are discussed in more detail by Gruber et al. (2009). Also, the temperature shift, which is necessary in germ-line mutants, is subject of discussion in this excellent overview. In studies collected in the database, 79% of the wild type nematodes were maintained at the standard temperature of 20 °C, and 12% at 25 °C, which enables a faster performance of several tests (data not shown).

In most conditions, test compound concentrations between 11–100 µM or 11–100 µg/mL were used (Fig. 4d), whereas in total, concentrations from the nanomolar to molar range are covered. Despite this great variety, more than three-quarter of the compounds were tested only with one to three concentrations (Fig. 4e). Low-dose effects such as hormesis (Sun et al. 2020) as well as potential toxic effects at higher concentrations may remain unnoticed when using such a small range of concentrations. The hormesis effect is the biphasic response to low and high doses of chemical, biological, or physical exposures (Kendig et al. 2010), and is responsible for the known beneficial effects of toxins or dangerous radiation in low doses (Feinendegen 2005; Lajqi et al. 2019). However, several studies suggested that hormesis is also the main mechanism underlying the beneficial effects of natural extracts and polyphenols (Brunetti et al. 2020; Di Rosa et al. 2020; Martel et al. 2019; Saul et al. 2011; Sayed et al. 2021). This assumption is sometimes also called more specifically ‘xenohormesis’ (Lamming et al. 2004; Suter and Lucock 2017), and can be explained by the fact that certain plant metabolites, such as polyphenols, are produced especially in stressed plants (Mellway et al. 2009). The consumer of these plants might “interpret” the polyphenols as a warning signal for upcoming stress and thus, preventively activates its stress response, which eventually leads to enhanced life- and healthspan.

In the database, a relation of used concentrations and observed phenotypes can be observed, for instance, for the hexane extract of *Bacopa monnieri*, which increased the maximum lifespan at 1–10 µg/ml, and decreased it at > 50 µg/ml in the same study (Online Resource ESM_3). Thus, while a single concentration may be defensible for an initial (high-throughput) screen due to practical reasons, full dose–response curves are recommended for any compound flagged up during such a first-pass screen.

Regarding the *C. elegans* strain, 172 different ones were recorded in the database. In almost half (46%) of the 2995 analyzed conditions, the wild type strain N2 (var. Bristol) was used (Fig. 5a). Mainly to determine the involvement of certain proteins in the mode of action of the test compound, mutant strains were used in 28% of the conditions (Fig. 5a, b). One-fourth of the mutant strains lack *daf-16*, which is part of the prominent aging-related insulin-like signaling pathway (Murphy and Hu 2013). Its frequent use might be one of the reasons why *daf-16* is so often found to be (at least partly) responsible for beneficial effects of compounds in *C. elegans* (in 52% of the studies in the database which suggest a potential molecular background of compound action, DAF-16 was found to be (partly) responsible). Furthermore, 26% of the strains used have transgenes (Fig. 5a, c), which enable the localization and quantification of certain proteins, or the observation of the activity of promotors (indicated by “p” in the respective lines) directly in the transparent organism thanks to fluorescent labelling. Again, DAF-16 is one of the most common targets. Moreover, certain human diseases, like Alzheimer’s or Parkinson’s disease, can be modelled in *C. elegans* transgenic strains by incorporating human disease-relevant genes, with Alzheimer- and Huntington-models being the most popular transgenic organisms (Fig. 5c).

### Database searches for most promising compound candidates

Via the references to PubChem, the information on healthspan in our database is linked to chemical structures and to the wealth of biochemical insights represented in cheminformatics. Experiments can thus be clustered according to the structural similarity of compounds and to common structural motifs defining pharmacophores, to select interesting compounds for further testing.

**Fig. 6:**
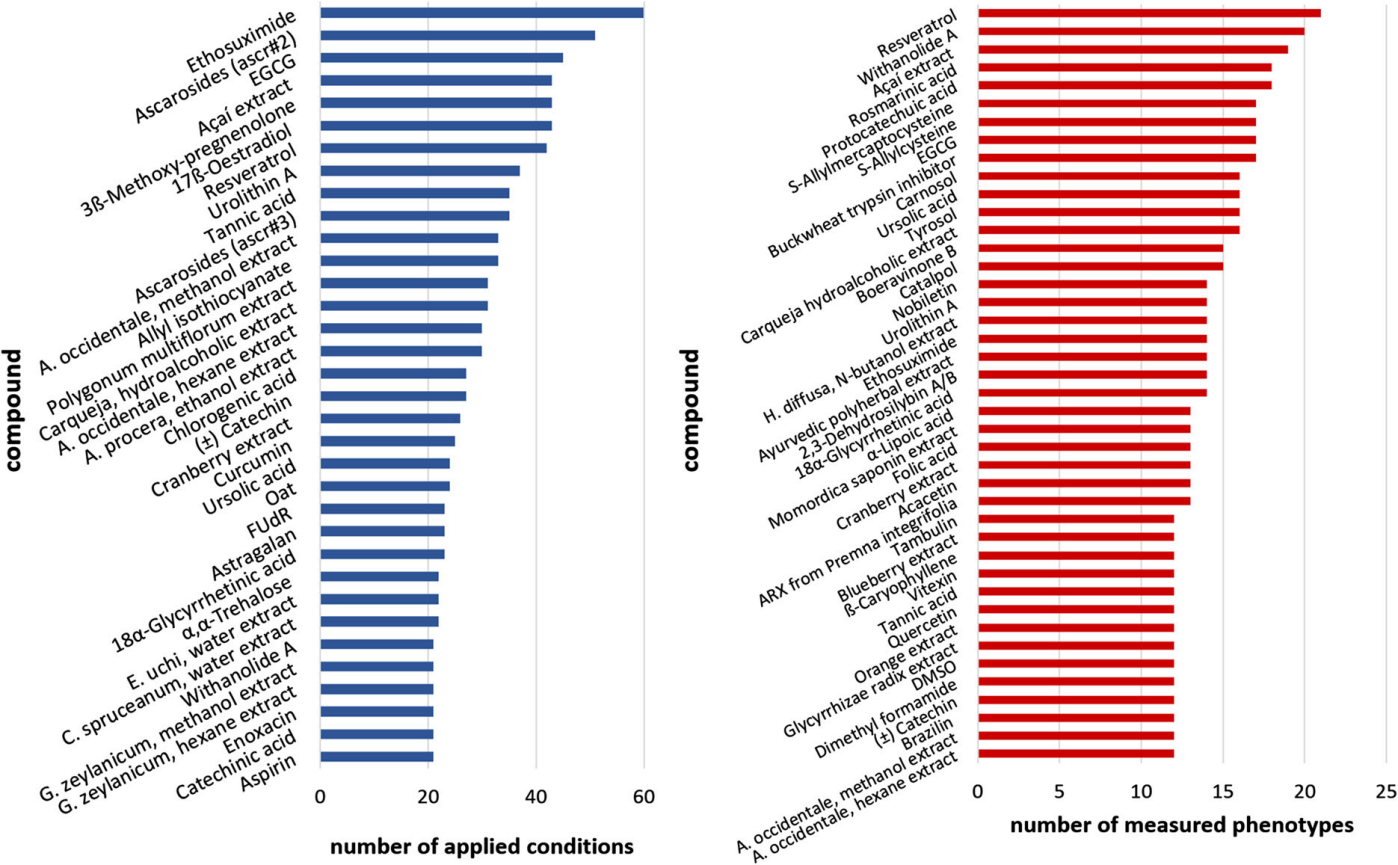
Most-studied compounds. The number of conditions were counted for each compound. Compounds investigated for more than 20 different conditions are shown (blue bars on the left). Furthermore, the number of observed phenotypes per compound was counted. The compounds with at least 12 different tested phenotypes are shown (red bars on the right)

In Fig. 6, compounds and the corresponding most-frequently studied conditions and phenotypes are displayed. Thirtyfour different compounds were studied in more than 20 conditions, which differ mostly by *C. elegans* strain and compound concentration. Moreover, 42 compounds were identified which were tested for at least 12 different phenotypes.

These compounds include some prominent polyphenols such as the racemic mixture of ( ±)-catechin, EGCG (epigallocatechin gallate), curcumin, quercetin, tannic acid, as well as resveratrol and one of its metabolites, piceatannol (which was tested in 20 conditions, as seen in Online Resource ESM_6, thus not displayed by the graph). The latter compound was shown, besides several natural extracts and Traditional Chinese Medicine formulas, to be the most effective heat stress-resistance enhancer in the database. Piceatannol at 100 µM could increase the survival during heat stress by an impressive 360% (Online Resource ESM_3). Similar benefits were observed for piceatannol during oxidative stress, though 220 µM EGCG also showed strong effects on this phenotype, with an increase of survival by 289%. Regarding physical function, again, piceatannol showed one of the strongest activities by increasing locomotion on solid media by 60%.

The largest number of conditions pertained to treatments with the anticonvulsant “ethosuximide” (Fig. 6), which regulates neural activity and is used to treat epilepsy in humans. These 60 different conditions were collected from only three different publications and comprise 10 ethosuximide concentrations, three temperatures, two exposure timings, three food qualities, and 10 different *C. elegans* strains (Online Resource ESM_3). All assays were performed on NGM agar, and most conditions included FUdR and ampicillin as additives. Thirty mM ethosuximide (about 4 mg/mL) was shown to promote longevity in wild type by increasing mean, median and maximum lifespan by about 30% in a temperature-dependent manner; 2 mg/mL triggered a delay in reproductive aging and enhancements in physical functions. Interestingly, UV-irradiated ethosuximide exerted life-shortening effects at the same concentration through an unexpected photochemical conversion into an unknown toxic compound (Choi et al. 2013).

The largest number of phenotypes was measured after resveratrol treatment (Fig. 6). This polyphenol attained fame by its diverse putative health effects together with its presence in red wine. Resveratrol features antioxidant, anti-inflammatory, immunomodulatory, and glucose- as well as lipid-regulating activities. Its preventive usage in humans has been suggested for numerous diseases, such as cancer, cardiovascular and neurodegenerative diseases, liver diseases, obesity, and diabetes (Meng et al. 2020). However, its clinical value was questioned by Vang et al. (2011), Sahebkar et al. (2015), Bo et al. (2016), and Berman et al. (2017). In our database, resveratrol data were collected from nine publications covering 21 phenotype measurements and 42 conditions. The application of 50 – 200 µM resveratrol resulted in enhanced survival during oxidative stress in young adult worms, and in lifespan prolongation (Online Resource ESM_3). However, Chen et al. (2013) observed life-prolonging effects only in high-glucose conditions. Furthermore, heat stress-resistance in young adult worms was influenced in positive as well as negative directions, or not at all, depending on the applied conditions. Moreover, Gruber et al. (2007) reported that the beneficial effects of resveratrol were accompanied by a reduction of fecundity. Thus, the beneficial effects of resveratrol are not universal, but very much dependent on the applied condition and on the measured phenotype.

## Discussion

### Phenotypic measurements: caution with antioxidative capacity, ROS, autofluorescence and lipid content

SODs and CTLs are well known antioxidant enzymes (Ighodaro and Akinloye 2018). Thus, by measuring their abundance or activity, the antioxidative capacity of the worm is supposedly evaluated. However, as discussed by Costantini and Verhulst (2009), it is not clear whether higher antioxidative capacity indicates a response to elevated oxidative stress or, in contrast, indicates an accumulation of unused antioxidants due to decreased stress. A detailed analysis of the whole organisms’ oxidative stress status, enzyme activities, as well as non-enzymatic metabolites would be necessary to interpret the measured capacities correctly. But only 35% of the SOD measurements collected in the database were accompanied by measurements of the total oxidative stress level. In contrast, for catalase assays the oxidative stress level was measured in parallel in 77% of the cases. Thus, (anti-)oxidation-related data must be interpreted cautiously.

Furthermore, the execution and interpretation of the measurements of the oxidative stress level are debatable. First, the link between ROS and aging is not clear. The causal relationship of ROS and aging was summarized and justifiably questioned by, amongst others, Back et al. (2012a), Van Raamsdonk and Hekimi (2010) and Gems and Doonan (2009). Furthermore, considering mitohormesis, beneficial effects of ROS have been suggested (Ristow 2014; Ristow and Schmeisser 2014). The term “mitohormesis” was first introduced as a hypothetical concept by Tapia (2006), and was experimentally underpinned shortly thereafter by Schulz et al. (2007). This hypothesis is based on the assumption that the response to mild mitochondrial (thus, oxidative) stress is beneficial, and was further underlined by the finding that some antioxidative capacities do not affect nematode lifespan (Pun et al. 2010). Thus, a decrease in the level of ROS does not necessarily indicate a health-promoting effect. Second, endogenous oxidative stress was mainly determined by the indirect detection of ROS using the fluorescent indicator 2,7-dichlorodihydrofluorescein diacetate (H_2_DCFDA). Further methods included the determination of carbonylated proteins, thiol level, and the lipid peroxidation markers 4-hydroxynonenal (4-HNE), F3-isoprostane, and malondialdehyde (MDA) in the animals. Labuschagne and Brenkman (2013) as well as Dikalov and Harrison (2014) provide an excellent overview of the different methods to determine endogenous oxidative burden. Despite its frequent use, the H_2_DCFDA assay was found to be unsuitable to measure intracellular ROS in *C. elegans*, which was affirmed by the editorial board of the journal “Free Radical Biology and Medicine” (Dikalov and Harrison 2014). Several shortcomings were also detected for other single approaches. Thus, Labuschagne and Brenkman (2013) recommend a combinatorial approach by using transgenic strains bearing fluorescent biosensors, such as the H_2_O_2_-sensor “HyPer”, the glutathione-sensor “Grx1-roGFP2”, or the redox proteomic technique “OxICAT” (Back et al. 2012b; Knoefler et al. 2012) in combination with measurements of protein carbonylation, DNA damage, or lipid peroxidation.

Two further measurements with possible pitfalls are autofluorescence and triglyceride/fat/lipid content. Autofluorescence is a well-known biomarker of the aging status in *C. elegans*, and is mainly found in intestinal intracellular granules of lysosomal origin. Its role as an indicator of lipofuscin, which is known from mammalian cells, was cast into doubt by Pincus et al. (2016), who concluded that a complex mixture of molecules is responsible for autofluorescence in *C. elegans*. Furthermore, they showed that autofluorescence is a reliable aging biomarker in *C. elegans* when using red fluorescence, but not blue or green fluorescence (Pincus et al. 2016). Unfortunately, the vast majority of studies were performed with the latter two types of fluorescence (green fluorescence: 28.9%, blue fluorescence: 67.5%, and red fluorescence: 3.6%; Online Resource ESM_6). Blue fluorescence is also described as “death fluorescence” due to its remarkable increase right before and during the dying process, where anthranilic acid was held responsible for this effect (Coburn and Gems 2013). Moreover, also the determination of the lipid content of nematodes is subject to intense criticism. In 2009, O'Rourke et al. (2009) could show that the commonly used Nile red staining is not suitable to determine the total lipid content in *C. elegans*. Thus, the results obtained from measuring these biomarkers should be treated with caution.

### Age at compound exposure and phenotypic measurements matters

Depending on temperature and culture conditions, nematodes reproduce until the 4th–8th day of adulthood, and the first deaths usually do not occur before the 7th–10th day of adulthood (in the following also abbreviated by A7-A10). Thus, since the effect of a compound is often measured during the first adult days, when symptoms of aging are largely absent, this does not provide much suitable information regarding its potential aging-deceleration effects. However, it is debatable which time point might be the best one to study those effects. Especially when taken into account that survival of the nematodes in the wild is very low compared to worm populations grown in the lab (with only a few individuals reaching the end of their reproduction period), the time after reproduction ceased might be considered “dying” rather than “aging” (Lohr et al. 2019). Unfortunately, few studies in *C. elegans* document the effects of compounds on the length of the reproductive period. It seems logical for aging studies to score healthspan parameters from the time they start declining. Treatment should then logically start at or before that time if one aims for maximum preventive effect. In studies for reversal of some aging phenotype, on the other hand, effects would appear the more attractive the later in life they can be shown, and the stronger they reverse the aged phenotype.

The importance of using aged worms for phenotypic measurements is also underlined by those studies in the database which use different age groups for the test performance. The compound treatments, for instance, with 20 µM 4-hydroxy-E-globularinin (Shukla et al. 2012b), 300 µM gallic acid (Saul et al. 2011) or 50 µM urolithin A (Ryu et al. 2016), did not lead to significant changes in pharyngeal pumping in younger nematodes (age A1-A3), but they led to an enhanced pumping frequency in older worms (A5, A9, or A7/A14, respectively). Thus, in studies focusing on aging, the measurements solely in young *C. elegans* could lead to false negative results. Moreover, the studies with salicylamine and ginseng suggest that some aging-decelerating effects are not visible in all aged nematodes, but only in animals towards the very end of their lifespan: 100 µM and 500 µM salicylamine (Nguyen et al. 2016) and 100 µg/mL Wisconsin ginseng extract (Cao et al. 2007) increased pharyngeal pumping at A20 and A14, respectively, but not at A5-A15 or A5-A11, respectively.

There are further examples of age-at-measurement effects, with other phenotypes such as locomotion and the level of ROS. The locomotion in young nematodes (age A1-A4) did not benefit from treatments with 50 µM brazilin (Lee et al. 2017), 50 µg/mL dichloroacetate (Schaffer et al. 2011), 0.24 g/mL *Glycyrrhizae radix* extract (Ruan et al. 2016), 10 µM icariside II (Cai et al. 2011), or 100 µM protocatechuic acid (Kim et al. 2014), but the beneficial effects of these treatments were visible at older ages (A8, A8-A21, A12, A10, or A8, respectively). Similar observations were also reported in locomotion measurements using 50 µM or 100 µM piceatannol (Shen et al. 2017), 25 µM silymarin (Kumar et al. 2015), 25 µM tomatidine (Fang et al. 2017), and 200 mg/mL blueberry extract (Wang et al. 2018), showing positive effect of the treatments only in aged nematodes.

Contrariwise, we found also a few observations with beneficial effects in younger, but not in older nematodes. At 20 µM, 10-O-trans-p-coumaroylcatalpol increased pharyngeal pumping at A1, but not at A6 (Shukla et al. 2012a), whereas 50 µM ellagic acid, urolithin C, or urolithin D were able to increase the pumping rate at A7 but not at A14 (Ryu et al. 2016). Thus, by ignoring aged nematodes in measurements, false positive results could be found, in addition to the false negatives already described.

Finally, contrasting results between different ages were reported. For example, 500 µM myricetin decreased the locomotion at A1, but increased it at A9 and A10 (Jung et al. 2017). Furthermore, 0.1 µM arsenite increased the ROS-level at A1/A2, but decreased the level at A5 (Schmeisser et al. 2013). Similarly, 10 µM of a recombinant buckwheat trypsin inhibitor strongly increased the ROS-level by 60% at A1, whereas the level was decreased by 20–38% at A3-A7 (Li et al. 2015). Interestingly, the contrasting effects of this inhibitor in different age groups were also visible in other phenotypes, namely the GSH content as well as the activity of catalase and SOD. However, it remains an open question whether the different observations in different age groups are really based on the age and frailty of the nematodes on the day of performance testing. Another possible and plausible reason might be the different amount of time that elapsed between exposure to the compound and the performance measurement. Indeed, since most exposure scenarios start at the stage of L4/A1, a measurement at e.g. A3 is done after 2–3 days of treatment, whereas the compound has more time to elicit any effects when testing occurs after continuous compound administration at A10 or even later. On the other hand, if the compound is administered only once, and there is no monitoring of its pharmacokinetics, it may or may not be present at the time of phenotype measurements, and its effects may or may not have disappeared at the later stages.

However, not only the age at phenotypic measurement is crucial, but also the timing of compound exposures. It is a surprising finding that only 1% of the studies were performed with exposures starting older than A1, since most of the studies focused on the aging-related effects of the test compounds in *C. elegans* and are supposed to (or were claimed to have) predictive value for subsequent human studies. In humans, however, treatments with those compounds would rather start at advanced ages, and not in children or early adulthood. It was already shown that the time when treatment administration starts can have a major impact on the effect of a compound. The prominent health-and lifespan-prolonging abilities of metformin, for instance, were recently verified in *C. elegans* when starting the treatment at the 1st day of adulthood, but resulted in opposite effects when starting at the 10th day of adulthood (Espada et al. 2019). Interestingly, lifelong treatment with metformin slightly increased mean lifespan of female mice, but surprisingly decreased lifespan in male mice (Anisimov et al. 2010; Blagosklonny 2010). Moreover, the inclusion or exclusion of the larval development phase during compound treatment can make a crucial difference for the outcome of certain phenotypes, as shown by Guha et al. (2014). They used cranberry extract starting with the egg or last larval stage, and could observe an increased heat stress resistance and mean lifespan in both scenarios. However, the pathogen resistance against *Vibrio cholerae* and locomotion were only enhanced when the treatment started at the egg stage.

The application of different exposure scenarios as well as the use of different age groups during phenotype measurements was observed several times, not only between different labs, but also within the same study. To name a few, 10 µM S-allylcysteine was added to the nematodes starting at A1 (Ogawa et al. 2016). Thereafter, the ROS level, as well as heat and oxidative stress resistance were measured at A3, but food consumption at A8. Furthermore, proteasomal activity was measured at A2 when the S-allylcysteine exposure started at L1 in the same study. Another example is given by a Traditional Chinese Medicine (TCM) study where lifespan was monitored after an exposure start at L4, but stress resistance was determined after starting the exposure at L1 (Wan et al. 2014). In a last example, *C. elegans* was treated with 50–200 µg/mL of the water extract from *Calycophyllum spruceanum* starting at L1, and the oxidative stress resistance as well as ROS level were measured at L4. The body length and reproduction, which are often used to detect any negative side effects of a treatment, however, were determined after treatment initiation at L3/L4. Moreover, the quantity of carbonylated proteins and the pharyngeal pumping rate were measured at A5 or A10, respectively, after starting the treatment at A1 (Peixoto et al. 2018).

Naturally, different phenotypes sometimes require a different time setting. The measurement of autofluorescence as an age-pigment should be performed in old worms, whereas chemotaxis is hard to measure in very old animals due to the weak moving behavior likewise, reproduction can only be assessed in very young animals. However, in several cases collected in this database, the different selected exposure and measurement times seem to be avoidable or arbitrary. The above examples show that conclusions about treatment outcomes are often derived from inconsistently performed studies with varying time points for phenotype measurements as well as treatment initiation and duration (not to mention dose). However, to determine whether these inconsistencies lead to erroneous conclusions or not, a direct comparison of the different conditions would be necessary.

### Potential bacteria-compound interactions by using live OP50?

The selection of the bacterial food is of major importance for compound-based assays in *C. elegans*. Plant polyphenols and extracts, for instance, are often studied in *C. elegans*-based healthspan assays, and were shown to have diverse antibacterial effects (Coppo and Marchese 2014). By adding these compounds to the alive feeding bacteria or growth medium in order to test their activities in *C. elegans*, a compound-bacteria interaction cannot be excluded. Antimicrobial activity could inhibit bacterial proliferation, which in turn could reduce the harmful intestinal accumulation of bacteria during aging (Portal-Celhay et al. 2012). Furthermore, a moderate dietary restriction (DR) effect due to a compound-induced reduction of bacteria is also conceivable. These mechanisms could lead to false-positive results by simulating beneficial polyphenol-induced effects in *C. elegans*, which are, however, independent of the action in the target organism but are solely based on the compound-bacteria interaction (Choi et al. 2013). A few examples providing evidence for this assumption can be found for lifespan data. 50–800 µg/mL Alaskan chaga extract (Scerbak et al. 2016) as well as 300 µM gallic acid and 50 µM ellagic acid (Saul et al. 2011) extended nematode mean lifespan in the presence of live OP50, but not of heat- or UV-inactivated bacteria. 4 mg/mL ethosuximide prolonged mean lifespan when using dead or live bacteria; however, maximum lifespan could only be enhanced when using live OP50 (Evason et al. 2005). At least in terms of lifespan, these treatments seem to act mainly via antibacterial effects, which are usually not the focus in the search for health-promoting compounds. Furthermore, evidence for the involvement of bacterial metabolism in the health- and lifespan promoting abilities of compounds was also reported for metformin (Cabreiro et al. 2013) and diaminodiphenyl sulfone (Choi et al. 2019). Finally, Gusarov et al. (2013) provide evidence that a microbial metabolite, namely nitric oxide, is itself able to trigger health- and lifespan benefits. Thus, to avoid erroneous conclusions, providing food consisting of heat- or UV-inactivated bacteria could be helpful. It should be noted that more feeding than usual is necessary when using these non-proliferating bacteria in order to avoid food shortage.

The inhibition of bacterial proliferation is also possible by using antibiotics like carbenicillin, ampicillin or streptomycin, in order to minimize compound-bacteria interactions. However, the majority of the (rarely used) antibiotics were applied in combination with antibiotic-resistant feeding bacteria with the sole purpose of preventing contaminations. It should also be pointed out that bacteria may metabolize test compounds, thereby creating novel ones and reducing the concentration of the applied test compound.

Nevertheless, feeding *C. elegans* with killed bacteria influences its life- and healthspan depending on the bacterial killing-method (Lenaerts et al. 2008). Furthermore, the microbiome-compound interaction might also be important for several beneficial compound-effects in humans, thus, the microbiological environment should be carefully selected for *C. elegans* studies. Indeed, metformin mediated enhancement of life- and healthspan is a very valuable result and would be probably overlooked when studying *C. elegans* with dead bacteria only (Cabreiro et al. 2013; Cabreiro and Gems 2013). The optimal performance of compound studies in *C. elegans* would probably require their natural food source as described in Khan et al. (2018). This would equip the worms with their natural microbiome and thus, would be much better comparable to the complex relationship of the microbiome, food, host and pathogens found in humans. Since such a complex feeding regime for *C. elegans* would be quite demanding, to our knowledge yet no compound study implemented this.

### Jaccard similarity analysis highlights the diversity of compound mediated health changes

The pumping of the pharynx is strongly age-dependent in *C. elegans* (Collins et al. 2008) and it was shown that the function and structure of pharyngeal muscles are affected by aging-related decline, that is, specifically, sarcopenia (Chow et al. 2006). Thus, a higher pumping frequency is often regarded as a marker of decelerated aging. On the other hand, decreased pumping was also frequently linked to increased healthspan: exposure to the catechin metabolite ‘protocatechuic acid’ led to a decreased pumping rate in parallel to diverse health benefits such as increased stress resistance and locomotion as well as lifespan prolongation (Kim et al. 2014). Similar patterns were also observed for the antibiotic enoxacin (Pinto et al. 2018), folic acid (Rathor et al. 2015), the ethylacetate fraction of *Ribes fasciculatum* (Jeon and Cha 2016), and the iridoid glucoside ‘catalpol’ (Seo et al. 2015), to name a few. These contrary observations may arise because a lower pumping rate may not necessarily be just caused by aging-related processes, but it could in turn be a cause of a slowdown of such processes. The reduced pumping leads to reduced ingestion, which can then cause health- and lifespan benefits due to calorie restriction (Cypser et al. 2013). This could also be the reason for the low correspondence of pharyngeal pumping with other healthspan phenotypes observed in this survey.

The level of endogenous oxidative stress is strong negatively linked with heat stress resistance and locomotion. On the one hand, this is in line with the free radical theory of aging, which states that ROS are a cause of aging (Kirkwood and Kowald 2012). This theory suggests that an increase of the ROS level would accelerate aging, which results in health deterioration reflected in, amongst others, reduced locomotion and stress resistance. On the other hand, this clear link is also a bit surprising when taking into account that some oxidative stress can be beneficial for health via mitohormesis (Ristow 2014). There is no clear correspondence between the ROS level and pharyngeal pumping. Interestingly, the negative correspondence between the activity/quantity of catalases (CTLs) and ROS level is rather weak. This is consistent with the discussion by Costantini and Verhulst (2009), who explained that antioxidative power (e.g. provided by CTLs) and prooxidative burden (as indicated by the ROS level) can have numerous different distributions in organisms. Thus, a low level of ROS is not necessarily accompanied by high antioxidative capacity inside the organisms.

Our Jaccard similarity analysis suggests that the measurement of lifespan alone is rarely sufficient to define a compound’s action. Different healthspan parameters need to be addressed in order to get a full view of a compound’s capacity, since not all of them are sufficiently corresponding with each other. On the other hand, lifespan and stress resistance measurements seem to be interchangeable due to the observed high correspondence, which corroborates previous studies (Lithgow and Walker 2002). Adding a poorly corresponding parameter to an experiment will provide more information than adding an almost perfectly corresponding one.

### Strengths of compound-based healthspan studies in *C. elegans*

Despite the numerous gaps and weaknesses reported here, the “Healthy Worm Database” also highlights several strengths in compound-mediated healthspan studies in the nematode. First at all, the wealth of appropriate healthspan studies enables a targeted search for suitable compounds in mammalian and human studies. Due to the multitude of possible aging-decelerating treatments and due to the high expense in mammalian and human studies, a pre-selection of compound candidates is mandatory. Thus, the fast experimental turnover in *C. elegans* studies, as indicated by the wealth of conditions, phenotypes and compounds collected in this database, offers a good basis for compound selections.

Furthermore, *C. elegans* enables the unique opportunity to observe molecular changes in vivo by using different reporter strains. The database includes 69 different disease- or gene-specific transgenic strains, the majority with reporter proteins such as GFP or YFP. Thus, the effect of a compound on, for instance, the accumulation of human disease-relevant proteins such as α-synuclein is studied frequently as shown in the database. This allows the focused search for a disease-specific treatment.

Finally, despite all inconsistencies described in this study, which often hinder the direct comparison of results, some standards seem to be established. All studies made use of the same wildtype (N2) and almost three-quarter used alive OP50 as food, 20 °C as maintenance temperature and NGM agar as medium.

### Limitations of this study

All data presented in this database were manually collected. Thus, despite greatest care, it cannot be excluded that typos appear and that descriptions in the screened publications were misunderstood. Thus, everyone is encouraged to check the entries of their own (and other) publications and to inform us, when mistakes happened. Furthermore, the search phrase to select literature was carefully selected to retrieve a manageable number of hits. However, several papers which would be suitable for this study were not covered by this phrase. Thus, some of the conclusions made here are not covering the full set of available studies and are only made from a representative section of literature. Again, the community is encouraged to inform us about important missing studies, which will be incorporated thereafter. Another issue is the recency of the selected studies. The database comprises literature from 1993–2020. Thus, also studies with outdated methods influenced our results which therefore might not display the current situation accurately.

A further limitation, which should be mentioned here, is that most of the studies presented in the database were not performed in a blinded way. Blinding might not be necessary for automated measures, but it can increase the quality of manual measurements in which the researcher is directly involved. In manually performed lifespan and stress resistance analyses, for instance, researchers need to decide whether a worm is dead or alive by visual judgement. A certain expectation, such as an expected life prolonging ability of a substance, can have a substantial influence on this decision. Gruber et al. (2009) explained the operator bias in detail and suggest blinding and randomization especially for all survival studies in *C. elegans*. However, also other measurements included in our database, such as pharyngeal pumping, reproduction, body length, DAF-16-translocation, or locomotion, are strongly dependent on the researcher’s decision (except for automated measurements) and are thus, prone to operator bias. Since blinding was only rarely performed in the studies collected, many results might be not fully objective.

Finally, the targets of most single compounds in the database are unknown. And many entries describe extracts for which the functional compound is yet not described. This limits the immediate use of the data, or stimulates respective further insights into functional compounds with unknown targets. Especially for compounds/extracts with known effects for vertebrates, the investigation of molecular insights in a species with fewer ethical limitations and easy genetic modifications is valuable.

## Conclusion

The “Healthy Worm Database” provides an interactive tool for the search and selection of healthspan-promoting compounds in the model organism *C. elegans*. This data collection differs from other databases by focusing on compound-mediated healthspan effects instead of lifespan enhancements. Moreover, it enables a closer look on the aging research field by displaying its strengths and weaknesses, as well as remaining open questions. Further papers and compounds will be added to the database in the near future, and the creation of a weighted formula to determine the most effective compounds is in preparation.

## Supplementary Information

Below is the link to the electronic supplementary material.

Supplementary file1 ESM_1: Overview of biogerontology databases (PDF 175 KB)

Supplementary file2 ESM_2: Health-related measurements (PDF 174 KB)

Supplementary file3 ESM_3: Database flat file (XLSX 321 KB)

Supplementary file4 ESM_4: Criteria for the inclusion and exclusion of scientific papers (PDF 127 KB)

Supplementary file5 ESM_5: Screened publications (XLSX 917 KB)

Supplementary file6 ESM_6: Raw results (XLSX 68 KB)

## Data Availability

All data generated or analyzed during this study are included in this published article and its supplementary information files and are available at the Healthy Worm Database (http://healthy-worm-database.eu).
