# Supplementary material for: Health and longevity studies in C. elegans: the “healthy worm database” reveals strengths, weaknesses and gaps of test compound-based studies
Source: Biogerontology. 2021 Mar 8;22(2):215–36. doi: 10.1007/s10522-021-09913-2 (PMC7973913; doi:10.1007/s10522-021-09913-2)
Supplement: Supplementary file 1 — Supplementary file1 ESM_1: Overview of biogerontology databases (PDF 175 KB) [file 10522_2021_9913_MOESM1_ESM.pdf]

Title: Health and longevity studies in *C. elegans*: The "Healthy Worm Database" reveals strengths, weaknesses and gaps of test compound-based studies
Journal: Biogerontology
Authors: Nadine Saul, Steffen Möller, Francesca Cirulli, Alessandra Berry, Walter Luyten, Georg Fuellen
Corresponding author: Nadine Saul, Molecular Genetics Group, Institute of Biology, Humboldt University of Berlin, 10115 Berlin, Germany; Email: nadine.saul@gmx.de

ESM_1: Overview of biogerontology databases

Short title	Full title and link	Reference	Short description	Organisms included	Type of biogerontology relevant data
**AgeFactDB**	JenAge Ageing Factor Database (http://agefactdb.jenage.de)	(Hühne et al. 2014)	Lifespan data connected to genes, compounds or other factors such as dietary restriction	*H. sapiens* & different model organisms	Lifespan linked to different interventions in models and GWAS-data in humans
**AGEID**	The aging genes/interventions database (Formerly: https://omictools.com/ageid-tool; Now: http://www.uwaging.org/genesdb/)	(Kaeberlein et al. 2002)	A catalogue of lifespan experiments	*H. sapiens*, *M. musculus*, *D. melanogaster*, *C. elegans*, and *S. cerevisiae*	Mainly lifespan data related to gene-knockouts and knock-downs and GWAS-data in humans
**AGEMAP**	Atlas of Gene Expression in Mouse Aging Project (http://cmgm.stanford.edu/~kimlab/aging_mouse/)	(Zahn et al. 2007)	Changes in gene expression as a function of age in mice; downloadable datasheets only	*M. musculus*	Tissue-specific gene expression during aging
**AnAge**	The Animal Ageing and Longevity Database (http://genomics.senescence.info/species/)	(De Magalhaes and Costa 2009)	Featured by HAGR; Compilation of data on aging, longevity, and life history for comparative biology	*H. sapiens* and more than 4000 different species	Lifespan and life history data, no interventions
**CID**	CISBAN Interactomes Database (http://cisban-silico.cs.ncl.ac.uk/cid.html)	Developed by M. Taschuk, C. Simillion, J. Hallinan and A. Wipat	Cytoscape-based interaction data from model organisms used in aging research	11 model organisms, i.e. *C. elegans*, *H. sapiens*, *M. musculus*, *D. melanogaster*	Interactomes of genes
**DAA**	Digital Ageing Atlas (http://ageing-map.org/)	(Craig et al. 2014; Galperin et al. 2015)	Featured by HAGR; collection of age-related changes	*H. sapiens* & *M. musculus*	Tissue-specific molecular, physiological, psychological, and pathological changes related to aging
**Degradome**	The Mammalian Degradome Database	(Perez-Silva et al. 2016)	Collection of protease genes and their connection to diseases and aging	*H. sapiens*, *P. troglodytes*, and rodents	Mainly disease data linked to proteases

	(http://degradome.uniovi.es/dindex.html)				
**DrugAge**	The Database for Ageing-related Drugs (http://genomics.senescence.info/drugs/)	(Barardo et al. 2017)	Featured by HAGR; provides data on aging-related drugs across model organisms	30 different species; mainly *M. musculus, S. cerevisiae*, *C. elegans*, and *D. melanogaster*	Lifespan effects of compound treatments
**GenAge**	The Ageing Gene Database (http://genomics.senescence.info/genes/)	(de Magalhaes and Toussaint 2004)	Featured by HAGR; Database of genes related to aging	*H. sapiens* & 9 model organisms; mainly *M. musculus*, *D. melanogaster*, *C. elegans*, and *S. cerevisiae*	Mainly lifespan data related to gene knockouts and knock-downs, and GWAS-data in humans
**GenDr**	The Dietary Restriction Gene Database (http://genomics.senescence.info/diet/)	(Wuttke et al. 2012)	Featured by HAGR; Database of genes associated with dietary restriction	*M. musculus, S. cerevisiae*, *C. elegans*, *D. melanogaster, and S. pombe*	Mainly lifespan data related to gene knockouts and knock-downs
**Geroprotectors**	(http://geroprotectors.org)	(Moskalev et al. 2015)	Lifespan experiments with compound treatments	13 model organisms	Lifespan as influenced by compound treatments
**HAGR**	The Human Ageing Genomic Resources (http://genomics.senescence.info)	(De Magalhães et al. 2009; 2005; Tacutu et al. 2012; 2017)	Platform hosting different aging associated databases such as AnAge, DrugAge, GenAge, GenDR	See respective database	See respective database
**LibAge**	The Bibliographic Library for Ageing (http://libage.ageing-map.org/)	Developed by João Pedro de Magalhães	Featured by HAGR; Library for aging-related publications	Not determined	Aging-related literature
**MitoAge**	(http://www.mitoage.org/)	(Toren et al. 2016)	Database for comparative analysis of mitochondrial DNA	922 different species	Maximum lifespan linked to mitochondrial DNA sequence
**NeuroMuscleDB**	Neuro-Muscle Database	(Baig et al. 2019)	Genes associated with muscle development, neuromuscular diseases, aging, and neurodegeneration	*H. sapiens*, *M. musculus*, and *B. taurus*	Collection of muscle-related genes and connected phenotypes
**SynergyAge**	http://www.synergyage.info/	(Bunu et al. 2020)	Lifespan of animals which are modified by at least two genetic interventions	*D. melanogaster, M. musculus*, and *C. elegans*	Lifespan linked to genetic interventions

References:

Baig MH, Rashid I, Srivastava P, Ahmad K, Jan AT, Rabbani G, Choi D, Barreto GE, Ashraf GM, Lee EJ, Choi I (2019) NeuroMuscleDB: a Database of Genes Associated with Muscle Development, Neuromuscular Diseases, Ageing, and Neurodegeneration. Mol Neurobiol 56:5835-5843.

Barardo D, Thornton D, Thoppil H, Walsh M, Sharifi S, Ferreira S, Anžič A, Fernandes M, Monteiro P, Grum T (2017) The DrugAge database of aging ‐ related drugs. Aging Cell 16:594-597

Bunu G, Toren D, Ion CF, Barardo D, Sârghie L, Grigore LG, de Magalhães JP, Fraifeld VE, Tacutu R (2020) SynergyAge, a curated database for synergistic and antagonistic interactions of longevity-associated genes. Sci Data 7:366. doi:10.1038/s41597-020-00710-z

Craig T, Smelick C, Tacutu R, Wuttke D, Wood SH, Stanley H, Janssens G, Savitskaya E, Moskalev A, Arking R (2014) The Digital Ageing Atlas: integrating the diversity of age-related changes into a unified resource. Nucleic Acids Res 43:D873-D878

De Magalhaes J, Costa J (2009) A database of vertebrate longevity records and their relation to other life ‐ history traits. J Evol Biol 22:1770-1774

De Magalhães JP, Budovsky A, Lehmann G, Costa J, Li Y, Fraifeld V, Church GM (2009) The Human Ageing Genomic Resources: online databases and tools for biogerontologists. Aging Cell 8:65-72

De Magalhães JP, Costa J, Toussaint O (2005) HAGR: the human ageing genomic resources. Nucleic Acids Res 33:D537-D543

de Magalhaes JP, Toussaint O (2004) GenAge: a genomic and proteomic network map of human ageing. FEBS Lett 571:243-247

Galperin MY, Rigden DJ, Fernández-Suárez XM (2015) The 2015 nucleic acids research database issue and molecular biology database collection. Nucleic Acids Res 43:D1-D5

Hühne R, Thalheim T, Sühnel J (2014) AgeFactDB—the JenAge Ageing Factor Database—towards data integration in ageing research. Nucleic Acids Res 42:D892-D896

Kaeberlein M, Jegalian B, McVey M (2002) AGEID: a database of aging genes and interventions. Mech Ageing Dev 123:1115-1119

Moskalev A, Chernyagina E, de Magalhães JP, Barardo D, Thoppil H, Shaposhnikov M, Budovsky A, Fraifeld VE, Garazha A, Tsvetkov V, Bronovitsky E, Bogomolov V, Scerbacov A, Kuryan O, Gurinovich R, Jellen LC, Kennedy B, Mamoshina P, Dobrovolskaya E, Aliper A, Kaminsky D, Zhavoronkov A (2015) Geroprotectors.org: a new, structured and curated database of current therapeutic interventions in aging and age-related disease. Aging (Albany N Y) 7:616-628.

Perez-Silva JG, Espanol Y, Velasco G, Quesada V (2016) The Degradome database: expanding roles of mammalian proteases in life and disease. Nucleic Acids Res 44:D351-355. doi:10.1093/nar/gkv1201

Tacutu R, Craig T, Budovsky A, Wuttke D, Lehmann G, Taranukha D, Costa J, Fraifeld VE, De Magalhaes JP (2012) Human Ageing Genomic Resources: integrated databases and tools for the biology and genetics of ageing. Nucleic Acids Res 41:D1027-D1033

Tacutu R, Thornton D, Johnson E, Budovsky A, Barardo D, Craig T, Diana E, Lehmann G, Toren D, Wang J (2017) Human Ageing Genomic Resources: new and updated databases. Nucleic Acids Res 46:D1083-D1090

Toren D, Barzilay T, Tacutu R, Lehmann G, Muradian KK, Fraifeld VE (2016) MitoAge: a database for comparative analysis of mitochondrial DNA, with a special focus on animal longevity. Nucleic Acids Res 44:D1262-D1265

Wuttke D, Connor R, Vora C, Craig T, Li Y, Wood S, Vasieva O, Shmookler Reis R, Tang F, de Magalhães JP (2012) Dissecting the gene network of dietary restriction to identify evolutionarily conserved pathways and new functional genes. PLoS Genet 8:e1002834. doi:10.1371/journal.pgen.1002834

Zahn JM, Poosala S, Owen AB, Ingram DK, Lustig A, Carter A, Weeraratna AT, Taub DD, Gorospe M, Mazan-Mamczarz K, Lakatta EG, Boheler KR, Xu X, Mattson MP, Falco G, Ko MS, Schlessinger D, Firman J, Kummerfeld SK, Wood WH, 3rd, Zonderman AB, Kim SK, Becker KG (2007) AGEMAP: a gene expression database for aging in mice. PLoS Genet 3:e201. doi:10.1371/journal.pgen.0030201
