# Supplementary material for: Health and longevity studies in C. elegans: the “healthy worm database” reveals strengths, weaknesses and gaps of test compound-based studies
Source: Biogerontology. 2021 Mar 8;22(2):215–36. doi: 10.1007/s10522-021-09913-2 (PMC7973913; doi:10.1007/s10522-021-09913-2)
Supplement: Supplementary file 2 — Supplementary file2 ESM_2: Health-related measurements (PDF 174 KB) [file 10522_2021_9913_MOESM2_ESM.pdf]

Title: Health and longevity studies in *C. elegans*: The "Healthy Worm Database" reveals strengths, weaknesses and gaps of test compound-based studies
Journal: Biogerontology
Authors: Nadine Saul, Steffen Möller, Francesca Cirulli, Alessandra Berry, Walter Luyten, Georg Fuellen
Corresponding author: Nadine Saul, Molecular Genetics Group, Institute of Biology, Humboldt University of Berlin, 10115 Berlin, Germany; Email: nadine.saul@gmx.de

ESM_2: Health-related measurements

Phenotype Group	Phenotype	Number of compounds
**healthspan biomarkers**	acetylcholine level	1
**healthspan biomarkers**	activity / quantity / expression of CTLs	29
**healthspan biomarkers**	activity / quantity / expression of CYPs	4
**healthspan biomarkers**	activity / quantity / expression of GCS-1	2
**healthspan biomarkers**	activity / quantity / expression of glutathione peroxidase	4
**healthspan biomarkers**	activity / quantity / expression of GPDHs	1
**healthspan biomarkers**	activity / quantity / expression of GSTs	55
**healthspan biomarkers**	activity / quantity / expression of HSPs	62
**healthspan biomarkers**	activity / quantity / expression of SKN-1	1
**healthspan biomarkers**	activity / quantity / expression of SODs	66
**healthspan biomarkers**	activity / quantity / expression of UGTs	1
**healthspan biomarkers**	activity of acetylcholinesterase	4
**cognitive function**	aldicarb resistance	1
**healthspan biomarkers**	AMP:ATP ratio	1
**healthspan biomarkers**	antioxidative capacity	4
**physiological function**	arsenite stress resistance	1
**healthspan biomarkers**	ATP level	8
**healthspan biomarkers**	autofluorescence (age pigment / lipofuscin)	54
**healthspan biomarkers**	axonal dystrophy	3
**physical function**	body length / growth rate	52
**cognitive function**	chemotaxis	20
**healthspan biomarkers**	concentration of N(6)-Carboxymethyllysine (CML)	4
**physical function**	duration of defecation cycles	3
**healthspan biomarkers**	F3-isoprostane level	1
**physical function**	fast body movement span	6
**physical function**	fast pharyngeal pumping span	2
**physical function**	food consumption	5
**healthspan biomarkers**	GSH level	9
**physiological function**	heat stress resistance	202
**healthspan biomarkers**	hexokinase activity	1
**healthspan biomarkers**	intestinal bacteria colonization	1
**healthspan biomarkers**	lactate level	2
**healthspan biomarkers**	level of 4-HNE	1
**healthspan biomarkers**	level of autophagy	7
**healthspan biomarkers**	lipase activity	1
**lifespan**	maximum lifespan	103

**healthspan biomarkers**	MDA level	13
**lifespan**	mean lifespan	288
**cognitive function**	mechanical sensory / touch response	9
**lifespan**	median lifespan	76
**cognitive function**	memory	1
**physiological function**	metal stress resistance	1
**lifespan**	minimum lifespan	10
**healthspan biomarkers**	mitochondrial content/quality	5
**healthspan biomarkers**	mitochondrial DNA (mtDNA) integrity	1
**healthspan biomarkers**	mitochondrial DNA copy number	1
**healthspan biomarkers**	mitochondrial membrane potential	2
**healthspan biomarkers**	mitochondrial ROS production	1
**healthspan biomarkers**	muscle integrity	5
**cognitive function**	neuronal survival	7
**reproductive function**	number of offspring	92
**reproductive function**	onset of egg laying	9
**physiological function**	osmotic stress resistance	7
**physiological function**	oxidative stress resistance	213
**healthspan biomarkers**	oxygen consumption rate (OCR)	7
**physiological function**	pathogen stress resistance	7
**physical function**	pharyngeal pumping	91
**physical function**	pharyngeal total span	4
**healthspan biomarkers**	phosphofructokinase activity	1
**healthspan biomarkers**	phosphorylation level of PMK-1	1
**healthspan biomarkers**	proteasomal activity	9
**healthspan biomarkers**	pyruvate kinase activity	1
**healthspan biomarkers**	pyruvate level	2
**healthspan biomarkers**	quantity of apoptotic cells	2
**healthspan biomarkers**	quantity of carbonylated proteins	6
**reproductive function**	quantity of dauer formation	4
**cognitive function**	quantity of D-type GABAergic motorneurons	1
**reproductive function**	quantity of early reproduction	17
**reproductive function**	quantity of embryonic lethality	1
**healthspan biomarkers**	quantity of germline stem cells	1
**reproductive function**	quantity of internal hatch	1
**reproductive function**	quantity of late reproduction	12
**healthspan biomarkers**	quantity of polyQ aggregates	11
**healthspan biomarkers**	quantity of ß-amyloid	16
**healthspan biomarkers**	quantity of α- synuclein	16
**reproductive function**	reproductive growth span	1
**reproductive function**	reproductive total span	10
**healthspan biomarkers**	ROS level	113
**physiological function**	salt stress resistance	1
**reproductive function**	speed of larval development	13
**lifespan**	survival at a given time point	3
**healthspan biomarkers**	thiol level	2
**healthspan biomarkers**	translocation of DAF-16 to nucleus	73

**healthspan biomarkers**	translocation of SKN-1 to nucleus	16
**healthspan biomarkers**	triglyceride / fat / lipid content	27
**physiological function**	UV stress resistance	18
**physical function**	velocity / motility / body movement in liquid	24
**physical function**	velocity / motility / body movement on solid media	85
**reproductive function**	vertex (progeny per day)	21
