# Supplementary material for: Health and longevity studies in C. elegans: the “healthy worm database” reveals strengths, weaknesses and gaps of test compound-based studies
Source: Biogerontology. 2021 Mar 8;22(2):215–36. doi: 10.1007/s10522-021-09913-2 (PMC7973913; doi:10.1007/s10522-021-09913-2)
Supplement: Supplementary file 4 — Supplementary file4 ESM_4: Criteria for the inclusion and exclusion of scientific papers (PDF 127 KB) [file 10522_2021_9913_MOESM4_ESM.pdf]

Title: Health and longevity studies in *C. elegans*: The "Healthy Worm Database" reveals strengths, weaknesses and gaps of test compound-based studies

Journal: Biogerontology

Authors: Nadine Saul, Steffen Möller, Francesca Cirulli, Alessandra Berry, Walter Luyten, Georg Fuellen

Corresponding author: Nadine Saul, Molecular Genetics Group, Institute of Biology, Humboldt University of Berlin, 10115 Berlin, Germany; Email: nadine.saul@gmx.de

ESM_4: Criteria for the inclusion and exclusion of scientific papers

	Inclusion criteria	Exclusion criteria
**Language**	English	Non-English language of the full text
**Search engine**	PubMed	
**Publication type**	Short communications, research articles, book chapters	Conference posters or talks, protocols, reviews, newspaper articles, commercial reports, dissertations, master theses, bachelor theses, other student reports, videos
**Year of publication**	All	No digital full text or pdf file available
**Treatment**	Evaluation of at least one chemical treatment (natural or synthetic single compound, and mixtures as well as natural plant preparations, extracts or fractions)	Biological (all living organisms), physical (such as exercise, gravity, or radiation), genetic (such as gene knockout or knockdown), psychological/social (such as crowding level) treatments, or no treatment at all
**Organisms**	*Caenorhabditis elegans*	
**Observations**	• Physical function<br>• Physiological function<br>• Cognitive function<br>• Reproductive function<br>• Healthspan biomarkers	Only lifespan assays, toxicity tests, computational modelling, or demonstration of a new test system
